# AtbFinder Diagnostic Test System Improves Optimal Selection of Antibiotic Therapy in Persons with Cystic Fibrosis

**DOI:** 10.1128/jcm.01558-22

**Published:** 2023-01-05

**Authors:** G. Tetz, K. Kardava, M. Vecherkovskaya, A. Hahn, M. Tsifansky, A. Koumbourlis, V. Tetz

**Affiliations:** a Human Microbiology Institute, New York, New York, USA; b Division of Infectious Diseases, Children’s National Hospital (CNH), Washington, District of Columbia, USA; c Center for Genetic Medicine Research, Children’s National Research Institute, Washington, District of Columbia, USA; d Department of Pediatrics, George Washington University (GWU), Washington, District of Columbia, USA; e Divisions of Cardiac Critical Care Medicine and Pulmonary, Children’s National Hospital, Washington, District of Columbia, USA; f Division of Pulmonary and Sleep Medicine, Children’s National Hospital, Washington, District of Columbia, USA; g TGV-Dx, New York, New York, USA; Boston Children's Hospital

**Keywords:** cystic fibrosis, FEV, MDR, *P. aeruginosa*, antibiotics, pulmonary exacerbations

## Abstract

Cystic fibrosis (CF) is characterized by mutations of CFTR that lead to increased viscous secretions, bacterial colonization, and recurrent infections. Chronic Pseudomonas aeruginosa infection in persons with CF is associated with progressive and accelerated lung function decline despite aggressive antibiotic treatment. We report the management of respiratory infections in persons with CF with antibiotic therapy that was based on the recommendations of AtbFinder, a novel, rapid, culture-based diagnostic test system that employs a novel paradigm of antibiotic selection. AtbFinder mimics bacterial interactions with antibiotics at concentrations that can be achieved in affected tissues or organs and models conditions of interbacterial interactions within polymicrobial biofilms. This open-label, single-arm, investigator-initiated clinical study was designed to identify the efficacy of antibiotics selected using AtbFinder in persons with CF. Microbiological and clinical parameters were assessed following the change of antibiotic therapy to antibiotics selected with AtbFinder between January 2016 and December 2018 and retrospectively compared with clinical data collected between January 2013 and December 2015. We enrolled 35 persons with CF (33 with chronic P. aeruginosa colonization). Antibiotics selected using AtbFinder resulted in clearance of P. aeruginosa in 81.8% of subsequent cultures, decreased pulmonary exacerbations from 1.21 per patient per annum to 0, and an increase in predicted percent predicted forced expiratory volume in 1 s up to 28.4% from baseline. The number of systemic antibiotic courses used in patients after switching to the AtbFinder-selected therapy was reduced from 355 to 178. These findings describe the superiority of antibiotic regimens selected with AtbFinder compared with routine antimicrobial susceptibility testing.

## INTRODUCTION

Currently existing phenotypic and genotypic antimicrobial susceptibility testing (AST) methods are not always able to select effective antibiotics (1–4). These methods select for antibiotics predominantly based on antimicrobial sensitivity of only the lead pathogen isolated from a biological sample, which reflects the decades-old belief that infections are caused by a single pathogen (5). However, recent studies have shown that many human respiratory, urinary, skin, and soft tissue infections are polymicrobial and include complicated interactions between the pathogen and other bacteria, even if there is only a single causative pathogen (6–13). Given that conventional AST determines resistance and sensitivity to antibiotics of the leading pathogen in a very constrained, pure-culture condition that is completely different from the conditions at the site of infection, the effects of complex interbacterial interactions are not accounted for by existing AST methods (14–18). Failure to account for such interactions may have significant consequences, including (i) the development of collective antibiotic resistance within polymicrobial communities when the lead pathogens are protected by antibiotic resistance factors, such as efflux pumps, from other bacteria; and (ii) the unchecked (and untreated) activity of “bacteria helpers” or “accessory pathogens” that are required for the growth of lead pathogen (19–24).

Some studies have shown that within multispecies communities, certain factors secreted by one bacterium, such as Staphylococcus aureus protein A (SpA), alter multiple persistence-associated behaviors, leading to the persistence of Pseudomonas aeruginosa infection (7). In addition, recently published studies have shown that the joint response to antibiotics within a mixed community can be different (opposite) to that of the individual bacteria (25). Moreover, other bacteria at the site of the infection can often take the lead and promote the infectious process after the eradication of the lead pathogen.

The persistence of P. aeruginosa in persons with cystic fibrosis (CF) is another notable demonstration of the insufficient efficacy of ASTs in selecting effective antibiotic treatment (26). CF, a genetic disorder caused by a mutation in the cystic fibrosis transmembrane conductance regulator (CFTR) gene, is characterized by highly viscous mucus in the airways and chronic, polymicrobial respiratory infections (27). CF is a well-studied human disease associated with bacterial infection; therefore, persons with CF provide an excellent case in point for the study of antibiotic treatment on complex interbacterial interactions (28).

P. aeruginosa infection, a hallmark of lung function decline in persons with CF, is resilient to therapy and rarely eradicated, despite aggressive systemic and local antibiotic treatment (29–31). The persistence of P. aeruginosa in patients with CF is the result of not only the high antibiotic resistance profile of this pathogen but also the specificity of its behavior in polymicrobial communities that are not accounted for by routine ASTs (2, 14). We recently tested the efficacy of AtbFinder, a novel, culture-based test system developed to address the shortcomings of existing ASTs, including the issue of combined antibiotic resistance (32, 33).

AtbFinder is a rapid, phenotype-based system that introduces a novel principle of antibiotic selection wherein antibiotics are evaluated based on their efficacy against mixed biofilms cultured from the biological sample.

It is comprised of a multiwell plate filled with a novel TGV medium, with each well individually supplemented with antibiotics for the treatment of certain diseases. TGV medium was shown to allow culturing of a more diverse set of bacteria from polymicrobial biospecimens in the form of mixed biofilm, compared with that achieved with the standard media (32). Thus, antibiotics are selected based on their ability to modulate polybacterial cooperative interactions at the site of infection. Additionally, AtbFinder selects antibiotics based on concentrations achieved at the site of infection, unlike routine AST methods that utilize MIC and susceptibility breakpoints that reflect the pharmacokinetics of antibiotics in blood rather than in the tissue (34).

AtbFinder defines “effective” antibiotics by demonstrated bactericidal effect or inhibition of all bacteria growth in the specimen. More recently, we reported the case of a patient who, after renal cancer surgery, developed a recurrent urinary tract infection despite multiple antibiotic courses selected with antibiotics selected with conventional tests. The infection was successfully eradicated once antibiotics were selected with AtbFinder (35, 36). In the present study, we provide a prospective evaluation of the effectiveness of antibiotics selected with AtbFinder compared to a retrospective analysis of antibiotic efficacy selected with conventional culture-based AST in patients with CF, a paradigm disease for studying chronic, complex polymicrobial human infections (37).

## MATERIALS AND METHODS

### Study subjects.

This was a prospective, single-center, nonrandomized, open-label study utilizing AtbFinder to formulate an individualized antibiotic regimen in persons with CF. Study participants served as their own controls based on their clinical performance when treated for a prior period of time when the antibiotic regimen was determined using conventional AST (broth microdilution).

We analyzed 35 participants with CF, aged 15 to 59 years, who were recruited for the study from the Therapeutic Pulmonology Department, Scientific Research Institute of Pulmonology, Saint Petersburg Medical University, from 10 January 2013 to 31 December 2018. The whole study included 2 years before implementing antibiotic therapy selected using AtbFinder and marked as year −2 and year −1 (from 10 January 2014 to 31 December 2015) and 2 years after antibiotics were selected with AtbFinder and marked as year +1 and year +2 (from 10 January 2017 to 31 December 2018) (ClinicalTrials.gov identifier, NCT00437580). We intentionally did not include the data from year 0 in the present analysis of the clinical performance of antibiotics selected with different methods, as this was a transition year from the previous antibiotic treatment prescribed based on conventional ASTs to the treatment selected with AtbFinder. This year was excluded from analysis because patients transitioned to new antibiotic regimens at different times during this year and because some patients experienced pulmonary exacerbations before antibiotic selection using AtbFinder.

This study was approved by the Institutional Board Review (IRB) of the Saint Petersburg Medical University (PA-764/16, 2016) and followed the principles outlined in the Declaration of Helsinki. Each enrolled study participant who was over 18 years of age provided written informed consent, and for those study participants who were under 18 years of age written informed consent was provided by the participant’s legal guardian in addition to the child’s assent. During the prospective study period, the participant’s antibiotic therapy was switched from one selected with routine AST (broth microdilution) to those selected with AtbFinder, as described below. Antibiotic therapy guided by AtbFinder started after the enrollment of patients throughout 2016 and was monitored throughout 31 December 2018. During the study period, inhaled antibiotics were discontinued. Other medical treatments remained unchanged, with no mucolytics other than dornase alpha and/or hypertonic saline being used.

Study participants were eligible for enrollment if they had CF that had been diagnosed according to conventional criteria including a positive sweat chloride test (>60 mEq/L) and the identification of CFTR gene-related mutations, the ability to spontaneously expectorate sputum, and to reproducibly perform pulmonary function testing. The presence of P. aeruginosa infection was assessed using spontaneously expectorated sputum.

Patients were excluded if they were using immunosuppressive drugs, such as systemic corticosteroids, or were participating in other clinical studies. Study participants were assigned on a rolling basis for the study, and antibiotic selection with AtbFinder was conducted either during check-up visits or during hospitalization due to pulmonary exacerbation. Doctors received the list of antibiotics suggested as effective according to AtbFinder recommendations, and it was solely their decision on how many antibiotics and in what form to prescribe to the patients.

Access to the retrospective microbiological and treatment records for the patients enrolled in the study from 10 January 2013 to 31 December 2015 was approved by both the management of the department and the ethical committee. Antibiotic selection during years −2 and −1 was performed based on conventional AST. The MICs of antibiotics were determined by the broth microdilution method according to the Clinical and Laboratory Standards Institute (CLSI) guidelines with some modifications as previously discussed (38, 39). The plates were incubated for 48 h, and the visual MIC readouts were taken at 24 h, comparing the growth in the control antibiotic-free well, and additionally at 48 h.

For the second portion of our study, deidentified sputum samples from persons with CF whose concurrent clinical cultures grew Burkholderia cepacia complex (BCC) were provided by Andrea Hahn from Children’s National Hospital (Washington, DC, USA). IRB approval for routine collection of demographic and clinical data in persons with CF was obtained from Children’s National Hospital (Pro6781, 8 Dec 2015). Participants ≥18 years old provided written consent, and written parental consent was obtained for patients <18 years old. Assent was obtained from children between the ages of 11 and 17 years.

### Clinical parameters.

Clinical and microbiological parameters of study participants one and 2 years after antibiotics were selected with AtbFinder (marked as year +1, year +2) were compared with retrospective clinical data obtained 1 or 2 years before the switch of antibiotic therapy to be guided by AtbFinder. These retrospective data were taken as baseline and marked as years −2 and −1.

Lung function was measured with spirometry according to the American Thoracic Society criteria and converted to the percent predicted forced expiratory volume in 1 s (FEV1%) (40). Blood inflammatory markers (white blood cell count [WBC; 10e9/L] and C-reactive protein [CRP; mg/L]) were collected. Body mass index (BMI) was measured as the weight in kilograms divided by the square of the height in meters.

Baseline FEV1%, WBC, CRP, and BMI were defined as the last tests performed when the study participant was clinically stable (i.e., without systemic antibiotics) or as the mean of the measurements taken at years −1 and −2 when the person with CF was clinically stable.

The absolute changes in predicted FEV1%, WBC, CRP, and BMI from baseline to years +1 and +2 taken during regular checkup visits when the study participants were clinically stable (i.e., not on any systemic antibiotics) and not experiencing pulmonary exacerbations, were assessed.

The number of pulmonary exacerbations was defined as an increase in respiratory symptoms requiring hospitalization, and treatment with antibiotics was assessed for each study participant (41).

### Respiratory sample processing.

The collected respiratory samples from the prospective study were stored at 4°C for up to 8 h before sample processing. Spontaneous sputum samples were collected from sterile specimen cups and were homogenized by mixing 1:1 (vol/vol) with sterile normal saline, vortexed, and heated in a 37°C heated bead bath for 15 min.

For the evaluation of BCC growth on the TGV agar, we selected sputum samples with previously confirmed BCC. Sputum samples from the patients from Children’s National Hospital are routinely collected and stored through an IRB-approved Data and Biorepository. For long-term storage, they are homogenized by mixing 1:1 vol/vol with dithiothreitol (Fisher Healthcare) and sterile normal saline, vortexed, heated in a 37°C bead bath for 15 min, and then pelleted through centrifugation (12,000 *g* × 10 min). Supernatants and pellets were then stored separately at −80°C.

### Culture and bacterial identification.

For P. aeruginosa isolation, 50 μL of the sputum samples was inoculated onto MacConkey Agar or Cetrimide agar (Sigma-Aldrich, St. Louis, MO, USA) incubated at 37°C for up to 4 days. Lactose-negative colonies on MacConkey Agar were selected and then subcultured onto a 5% sheep blood agar (Fisher Scientific, Dickinson).

To identify the BCC, we used Burkholderia cepacia-selective agar and cultured according to laboratory recommendations at 37°C for 24 to 72 h. Bacterial isolates were identified by using Vitek-2 (bioMérieux, France) according to the manufacturer’s instruction with a Gram-negative identification card.

P. aeruginosa bacterial density was assessed by serial 1:10 dilutions in PBS (pH 7.0) and was performed by plating 100 μL of sputum mix onto MacConkey agar plates (Oxoid). These plates were incubated at 37°C aerobically for 48 h to isolate P. aeruginosa. Bacterial density was reported as log_10_ CFU per milliliter of sputum.

### Antibiotic selection with AtbFinder.

Twenty microliters of each biological sample was directly plated onto the agar of each well of several 12-well AtbFinder plates. In the 12-well plates used in this study, “testing wells” contained TGV nutrient medium (Human Microbiology Institute, NY, USA) with antibiotics (one antibiotic per well) selected as per current CF therapeutic guidelines and “control wells” contained antibiotic-free TGV medium (42). AtbFinder plates were incubated at 37°C and 5% CO_2_ for 24 h in a Sanyo MCO-19AIC incubator (Sanyo, Japan). Antibiotics (amoxicillin-clavulanic acid, amikacin, aztreonam, azithromycin, ceftazidime, ciprofloxacin, clindamycin, clarithromycin, ceftriaxone, colistin, ceftazidime-avibactam, cefotaxime, doxycycline, cefepime, gentamicin, imipenem, josamycin, levofloxacin, meropenem, metronidazole, ofloxacin, trimethoprim + sulfamethoxazole, tobramycin for intravenous use, ancomycin) were added to the TGV medium at the respective mean concentrations that could be achieved at the site of infection, according to literature data. AtbFinder plates were incubated for 4 h, 8 h, and 24 h (Table S1 in the supplemental material).

The presence of microbial growth was identified with the naked eye and confirmed with a stereoscopic microscope, magnification ×10. Microbial growth in any “testing well” indicates that in the pathological material, microorganisms are resistant to the antibiotic in that well. In this case, the antibiotic is categorized as “ineffective.” The absence of bacterial growth in any well indicates that the antibiotic present in that well kills or inhibits the growth of all bacteria in the biological specimen. Such an antibiotic is categorized as “effective.” The doctors were provided with the report containing the list of antibiotics categorized as “effective” or “ineffective” according to the AtbFinder.

For the evaluation of the performance of TGV agar compared with other nutrient-rich media or *Burkholderia*-selective agar, some AtbFinder plates were filled with antibiotic-free TGV agar, LB agar, Columbia agar, or Burkholderia cepacia-selective agar (all from Sigma-Aldrich, St. Louis, MO, USA). AtbFinder reading was performed at 4-, 8-, and 24-h intervals.

### Antibiotic selection with conventional AST.

During the study, from the same biosamples, along with the AtbFinder, P. aeruginosa was isolated and susceptibility testing was performed by broth microdilution and disk diffusion in the clinical microbiology lab. AST was performed using reference methods according to the CLSI guidelines as previously described.

### Statistical analyses.

A two-way analysis of variance comparison test was applied within the same data sets to test the difference between parameters at each time point. A nonparametric paired Wilcoxon signed-rank test was applied to analyze samples before and after the selection of antibiotics with AtbFinder.

GraphPad Prism version 9 (GraphPad Software, San Diego, CA, USA) or Excel 10 was applied for statistical analysis and illustration, if not stated differently. A *P* value of <0.05 was considered statistically significant.

## RESULTS

Study participant characteristics are shown in Table 1. Between January 2016 and December 2016, 35 patients with diagnosed CF were enrolled in this study. The mean age at the time of selection of antibiotics using AtbFinder was 28.3 years, with the oldest subject being 62 years old.

**TABLE 1:** Demographic and clinical characteristics of study participants^*a*^

Characteristic	Value
Demographic variabilities	
Total no. of patients (*n*)	35
Male	22
Female	13
Mean age (yr, range)	28.3
CF genotype (*n*)	
ΔF508/ΔF508del	7
ΔF508/other	15
Other	13
Anthropometric measurements	
BMI score mean	17.9
Underweight BMI score	27
Normal weight BMI score	8
FEV1% predicted (mean ± SD)	43.5% ± 16.4%
>70% (*n*)	3
>60% to <69% (*n*)	0
>50% to <59% (*n*)	8
>35% to >49% (*n*)	18
<35% (*n*)	6
Chronic Pseudomonas aeruginosa infection (*n*)	33

a BMI, body mass index; CF, cystic fibrosis; FEV1%, percent predicted forced expiratory volume in 1 s.

### Clinical outcomes.

**FIG 1:**
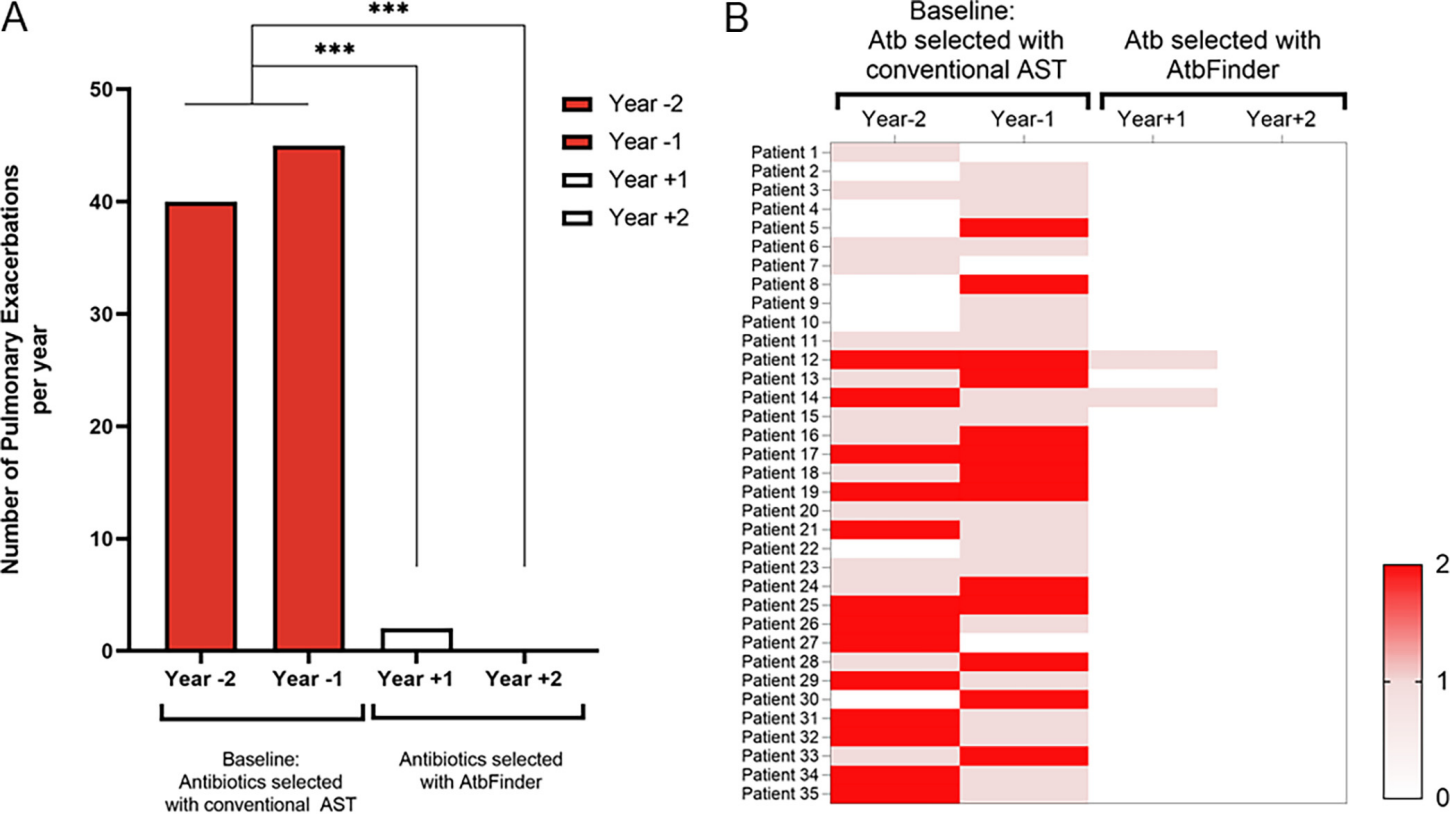
Effect of antibiotics selected with AtbFinder on the annualized rate of pulmonary exacerbations. (A) Number the overall estimated annualized pulmonary exacerbations leading to hospitalization treated with antibiotics selected with conventional AST or AtbFinder. ***, *P* < 0.001 compared to baseline years −1 and −2. (B) Heatmap of individual changes in pulmonary exacerbations from baseline through years +1 and +2. The number of pulmonary hospitalizations is represented by a color scale, from white (absence of pulmonary exacerbations) to red (maximum).

First, we found a significant reduction in the annualized rate of pulmonary exacerbations defined as a CF-related pulmonary condition requiring hospital admission, when patients switched to antibiotics selected with AtbFinder at year 0, compared to the time years −1 and −2 when antibiotics were selected using routine methods (Fig. 1A and B) (43). There was a reduction in the total number of annual hospitalizations due to pulmonary exacerbations in the studied group, from 45 to 40 in the baseline years −1 and −2, respectively, to 2 and 0 exacerbations in the years +1 and +2 of antibiotic selection with AtbFinder, respectively (all *P* < 0.001%). The estimated reduction in hospitalizations from the year before treatment selection with AtbFinder compared to the first and second years of treatment selection with AtbFinder was 95.6% and 100%, respectively (*P* < 0.001). Next, we examined changes in the number of hospitalizations for each patient and presented the data as a heat map (Fig. 1B). At the individual level, the mean annual rate of pulmonary exacerbations decreased from 1.21 per patient per annum at baseline to 0.057 during the first year of antibiotic selection with AtbFinder (*P* < 0.001). During the second year of antibiotic selection using the AtbFinder algorithm, there was a complete arrest of the development of pulmonary exacerbations with no hospitalizations required across the studied patients.

**FIG 2:**
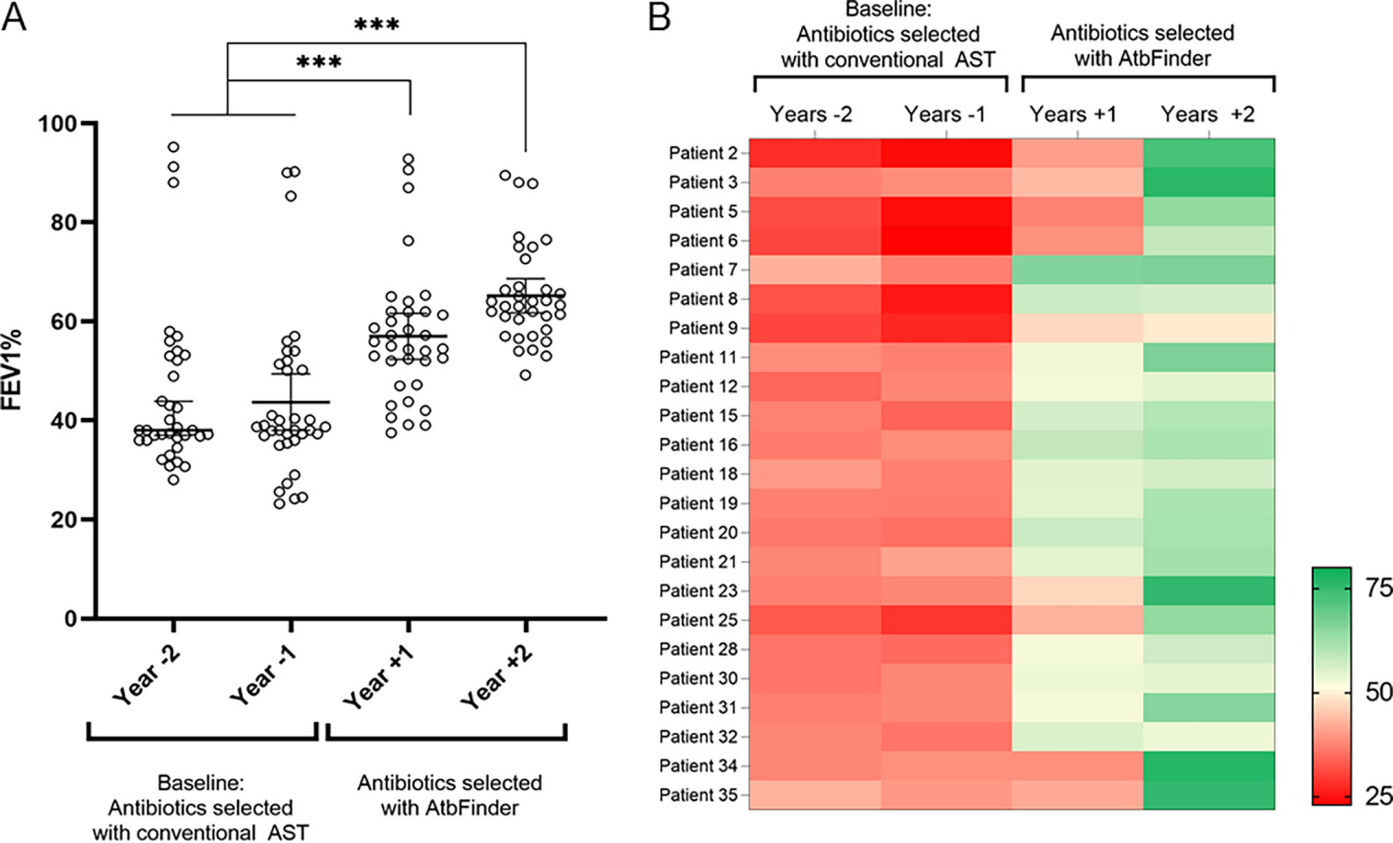
Dynamics of FEV1 (%predicted). (A) FEV1 (%predicted). Each dot reflects the measurement of FEV1% within each individual over a 1-year interval. After the use of antibiotics selected using AtbFinder, there was a significant increase in predicted FEV1 from 44.16% (SD ±16.33%) to 56.8% (SD ±13.30%) by year +1 and 65.1% (SD ±9.77) by year +2. ***, *P* < 0.001. (B) Heat map showing the dynamics of predicted FEV1% in patients categorized at the baseline as very severely or severely abnormal (predicted FEV1% from <35% to 49%). Red and green colors represent low and high percentages of predicted FEV1%, respectively.

We then observed the effect of antibiotics selected with AtbFinder on the predicted FEV1%, a common clinical assessment of lung function. For all patients, the mean predicted FEV1% at baseline year −1 was 43.5%. Treatment with antibiotics selected using AtbFinder resulted in a significant improvement in predicted FEV1% relative to the baseline period, with a mean absolute treatment difference of 12.7 percentage points (up to 56.9%) by year +1 and 20.9 percentage points (up to 65.1%) by year +2 (all *P* < 0.001) (Fig. 2A).

Next, we conducted a subgroup analysis of the 23 patients who at the baseline period were categorized as very severely or severely abnormal (with a mean FEV1 of 34.6% predicted; range, <35 to 49% predicted) to determine the effect of antibiotics selected using the AtbFinder within this group (Fig. 2B). We found that within 2 years following the change to antibiotics selected by AtbFinder, this parameter improved by 22.3 percentage points (up to FEV1 50% to 59% predicted) in 7 patients, by 28 percentage points (up to FEV1 60% to 69% predicted) in 10 patients, and by 39.2 percentage points (up to FEV1 >70% predicted) in 5 patients. The absolute change in the FEV% predicted in these patients was 16.4 percentage points (up to 51.1%) by year +1 and by 28.4 percentage points (up to 63.0%) by year +2 following the switch to antibiotic therapy selected using AtbFinder (*P* < 0.001). Taken together, implementing antibiotic therapy selected using AtbFinder resulted in an upward trend in lung function that had an average adjusted relative change of 41.0% per annum.

**FIG 3:**
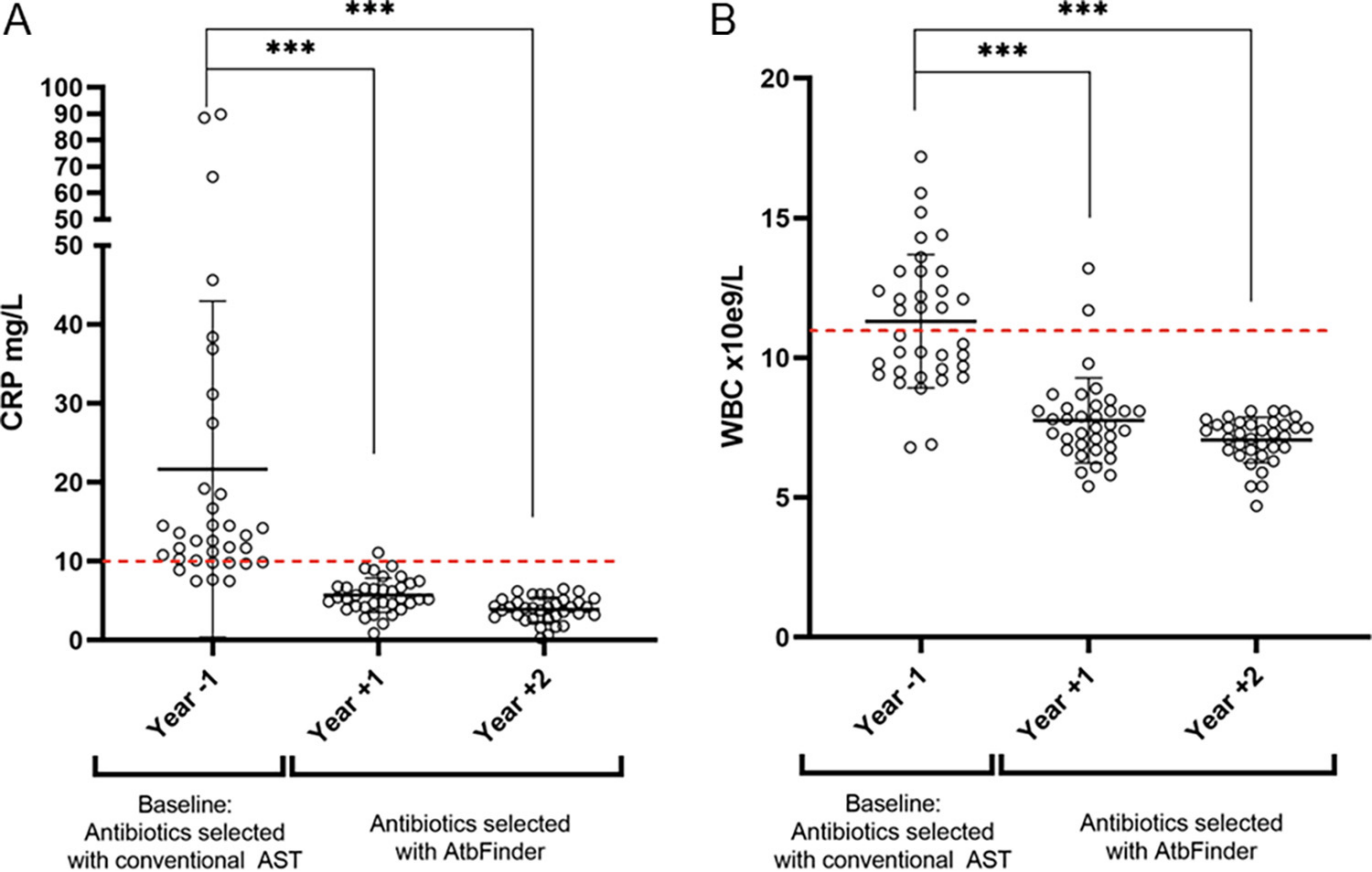
Effects of antibiotics selected with AtbFinder on inflammatory markers. (A) CRP levels before and after the use of antibiotics selected with AtbFinder. The red line represents the reference value the upper limit of normal (10 mg/L) (53). (B) WBC levels before and after the use of antibiotics selected with AtbFinder. The red line represents the upper reference value (11.0 × 109/L) (54). *, *P* < 0.05; **, *P* < 0.01; ***, *P* < 0.001.

Additionally, we evaluated how antibiotics selected with AtbFinder affected systemic, chronic inflammation based on the dynamics of inflammatory markers (WBC and CRP) measured during regular checkup visits when the patients did not experience pulmonary exacerbations. Both markers are known to correlate with lung injury in persons with CF. WBC and CRP levels were elevated in 33/35 and 34/35 patients in baseline year −1, respectively. There was a reduction of both inflammatory markers beginning in year +1, the first year of antibiotic selection with AtbFinder, compared to year −1 when antibiotics were selected using regular AST methods (Fig. 3A and B) (*P* < 0.001). Following treatment selected with AtbFinder, we observed a normalization of WBC in 19/35 patients in year +1 and 28/35 patients in year +2 (*P* < 0.001). We also observed normalization of CRP in 14/35 patients in year +1 and 26/35 patients by year +2 (*P* < 0.001).

### Microbiological outcomes.

We first observed that using AtbFinder, we were able to cultivate P. aeruginosa in 33 out of 35 patients, while standard methods identified P. aeruginosa only in 28 patients, meaning that with the AtbFinder we identified P. aeruginosa in 5 people who were previously believed to be P. aeruginosa free.

**FIG 4:**
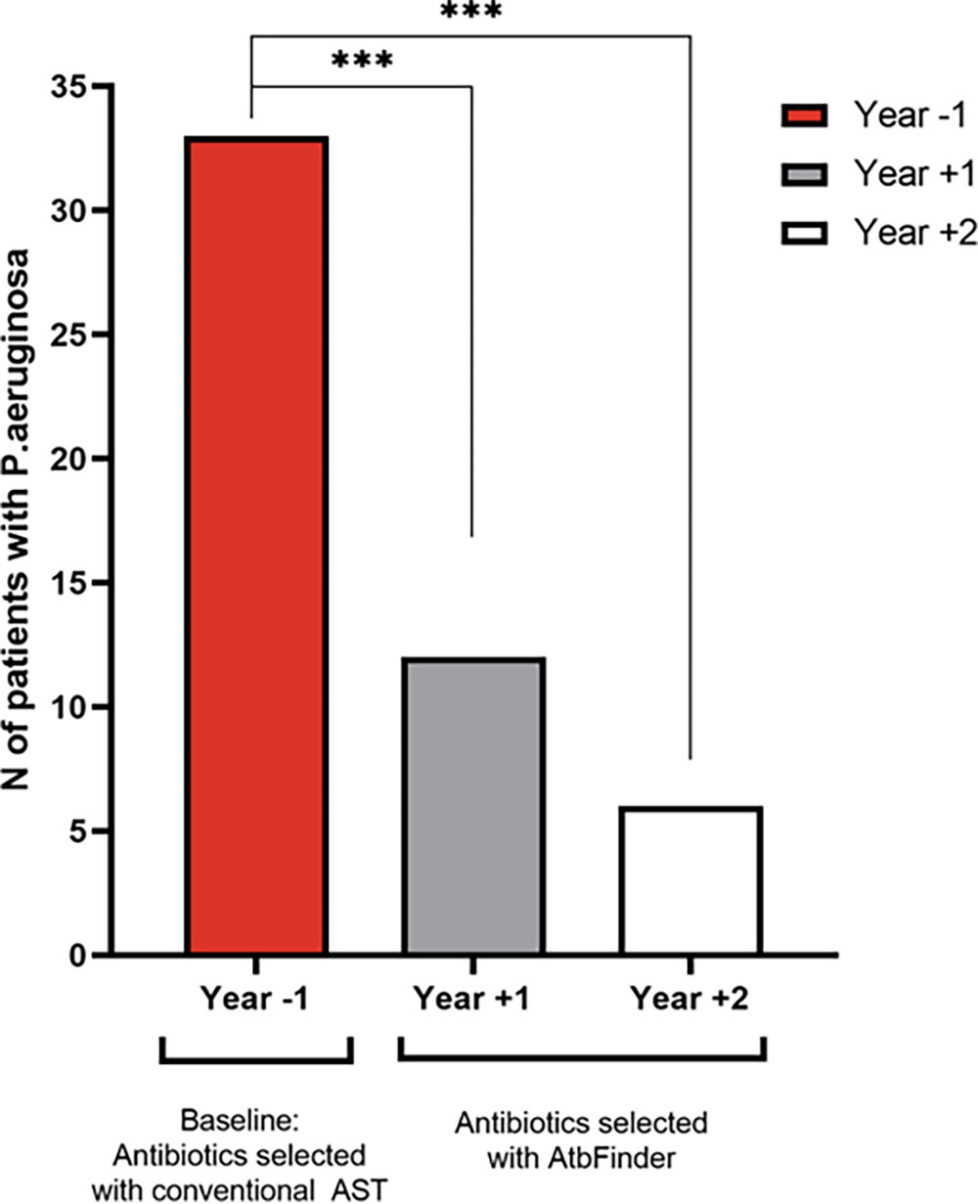
Number of individuals with a positive P. aeruginosa culture over time. Analysis was performed using Fisher’s exact test. ***, *P* < 0.001.

Next, we analyzed the effect of antibiotic therapy on P. aeruginosa culture clearance after changing antibiotic therapy from that selected based on standard methods to that selected with AtbFinder (Fig. 4). Specimens were collected and tested using routine culture methods to evaluate for the presence of P. aeruginosa every 3 months. All Pseudomonas-free intervals >6 months were considered culture negative. We found that, within a 2-year period, 81.8% of patients were cleared of P. aeruginosa (27 of 33 patients who initially tested positive for P. aeruginosa). By the end of the first year, P. aeruginosa was cleared in 63.6% (21/33 patients) and was cleared in an additional 18.2% (6/33 patients) by the end of the second year (*P* < 0.001). In addition, all 27 patients remained clear of P. aeruginosa for >24 months during the follow-up period and had 8 negative cultures (Table S2). Both groups whose respiratory cultures became P. aeruginosa culture negative and those who failed to clear P. aeruginosa with antibiotics selected with AtbFinder had very similar median ages of 27 years (range, 15 to 59 years) and 28 years (range, 24 to 38 years), respectively (Table S3). Moreover, there was no association between the length of colonization with P. aeruginosa and clearance success with antibiotics selected using AtbFinder. Thus, the median colonization time was 2.77 (±2.68) years in patients in whom P. aeruginosa was cleared and 2.83 (±1.95) years in patients in whom it has not been cleared from culture (Table S4).

### Particularities of antibiotics selection with AtbFinder.

**FIG 5:**
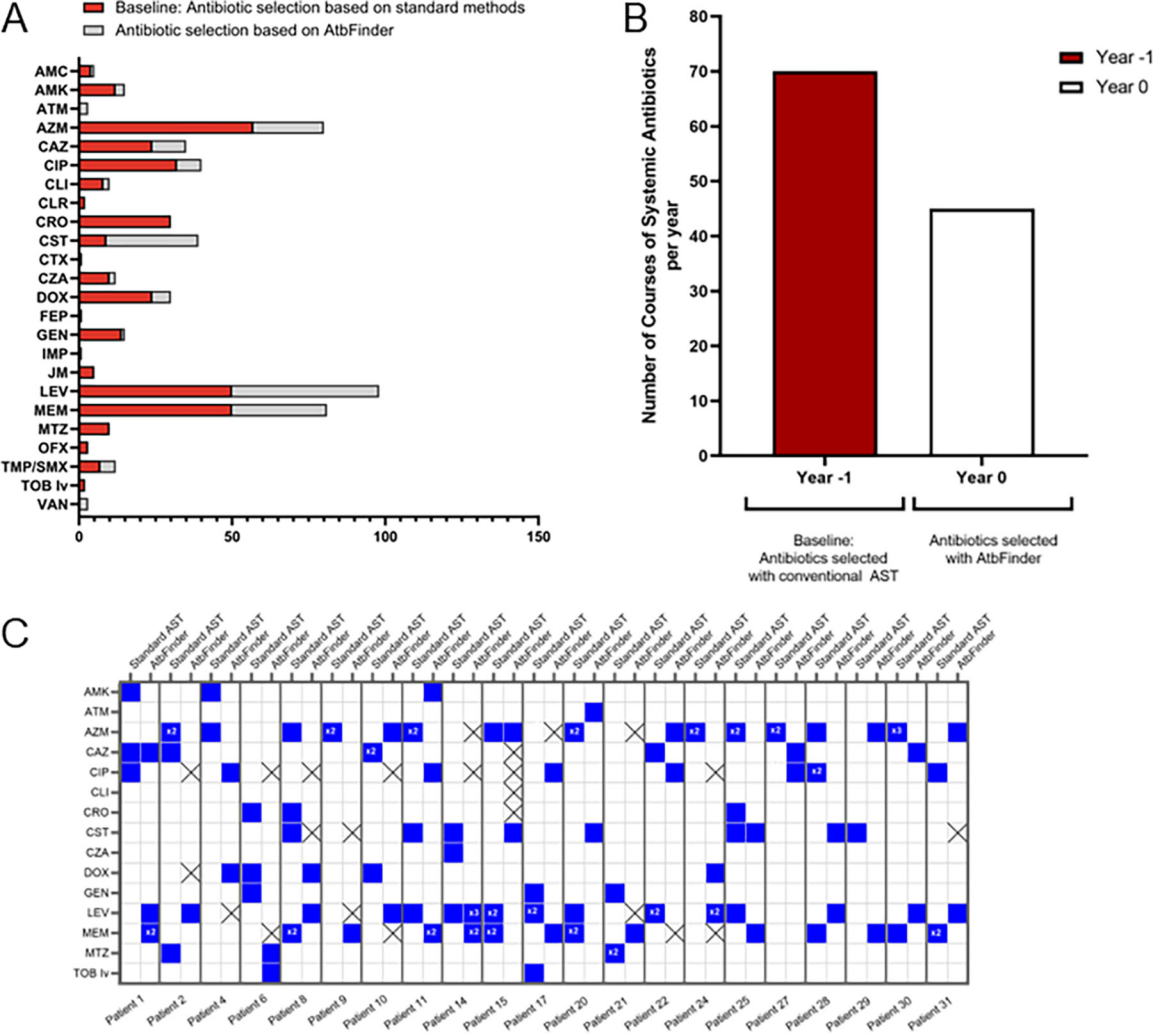
(A) The diagram reflects the total number of systemic antibiotic courses used based on selection with standard AST methods and with AtbFinder. Each column represents the total number of antibiotic courses used in 35 patients with CF during a 6-year period of which for 3 years antibiotics were used based on selection with conventional AST (red bar) and 3 years based on that by AtbFinder (gray bar). (B and C) Changes in antibiotic therapy following AtbFinder selection that led to P. aeruginosa clearance by the end of year 0. (B) Number of systemic antibiotics used per year in the baseline year −1 and the first year of antibiotic selection with AtbFinder year 0 that led to P. aeruginosa clearance. (C) Heatmap analysis with columns representing individual subjects who had clearance of P. aeruginosa by the end of the first year (year 0), following antibiotic selection with AtbFinder. The antibiotics used are on the left bar. The patient number and indicated analysis times period are on the top. “pre-” marks the antibiotics selected based on conventional AST at year −1; “post-”marks antibiotics selected with AtbFinder at year 0. The blue color key displays the antibiotics used in a particular patient. The number inside the blue squares indicates the number of therapeutic cycles this antibiotic was used during a year. The black cross marks are antibiotics suggested to be effective with AtbFinder but not selected for the treatment. AMC, amoxicillin/clavulanic acid; AMK, amikacin; ATM, aztreonam; AZM, azithromycin; CAZ, ceftazidime; CIP, ciprofloxacin; CLI, clindamycin; CLR, clarithromycin; CRO, ceftriaxone; CST, colistin; CZA, ceftazidime-avibactam; CTX, cefotaxime; DOX, doxycycline; FEP, cefepime; GEN, gentamicin; IMP, imipenem; JM, josamycin; LEV, levofloxacin; MEM, meropenem; MTZ, metronidazole; OFX, ofloxacin; TMP/SMX, trimethoprim + sulfamethoxazole; TOB iv, tobramycin for intravenous use; VNA, vancomycin.

After the completion of the study, we retrospectively compared which antibiotics were used in these patients before selecting antibiotics with AtbFinder. We found a striking difference in antibiotics selected as “effective” for the treatment based on conventional AST and those selected with AtbFinder. The first thing we noticed was the decrease in the number of systemic antibiotic courses from 355 during the 3-year period (baseline years −3, −2, −1) when antibiotics were selected with routine AST to 178 during a 3-year period (years 0, +1, +2) once patients were switched to the antibiotics selected by AtbFinder (Fig. 5A). There was a decrease in the total number of all broad-spectrum antibiotics used in patients after the antibiotics were selected with AtbFinder, with the exception of colistin, of which total use was increased from 9 antibiotic courses administered to 8 patients to 30 antibiotic courses administered to 14 patients.

Next, to evaluate the particularities of systemic antibiotic therapy guided by AtbFinder that led to P. aeruginosa clearance, we compared antibiotics selected with AtbFinder during the transition year 0 (which was the first year of implementing antibiotics selected with AtbFinder use) and those antibiotics used at baseline year −1 (based on the recommendation by standard AST) in 21 patients who were converted from P. aeruginosa positive to P. aeruginosa negative by the end of year 0 using antibiotics selected with AtbFinder (Fig. 5B and C). We first noticed a decrease in the overall number of antibiotic courses used, with 70 systemic antibiotic courses totally prescribed based on standard AST methods at baseline year −1 and 45 antibiotic courses based on AtbFinder recommendations at year 0 (Fig. 5B).

We also observed that the antibiotics identified as effective by AtbFinder were significantly different compared to those identified by standard AST (Fig. 5C). Therefore, the success of P. aeruginosa clearance was not the result of a higher number of antibiotics used but was due to the different antibiotic regimens selected using AtbFinder.

The three most common antibiotics used at baseline year −1 were azithromycin, followed by levofloxacin and meropenem. The most frequently used antibiotics after the use of AtbFinder were levofloxacin, meropenem, azithromycin, and colistin (Table 2). Fifty-seven percent of the study participants received combination systemic antibiotic therapy with beta-lactam plus another drug class at least once during the baseline year −1, and only 38% received such therapy after the use of AtbFinder in year 0.

**TABLE 2:** Comparison of the most frequently used antibiotics based on conventional AST recommendation at year −1 and AtbFinder at year 0

Antibiotic	No. of patients administered antibiotic (%)
Conventional AST at baseline year −1	
Azithromycin	12 (57.1%)
Levofloxacin	7 (33.3%)
Meropenem	6 (28.6%)
AtbFinder at year 0	
Levofloxacin	9 (42.6%)
Meropenem	7 (33.3%)
Azithromycin	5 (23.1%)
Colistin	5 (23.1%)

### Effect of the antibiotics selected with AtbFinder on body composition.

**FIG 6:**
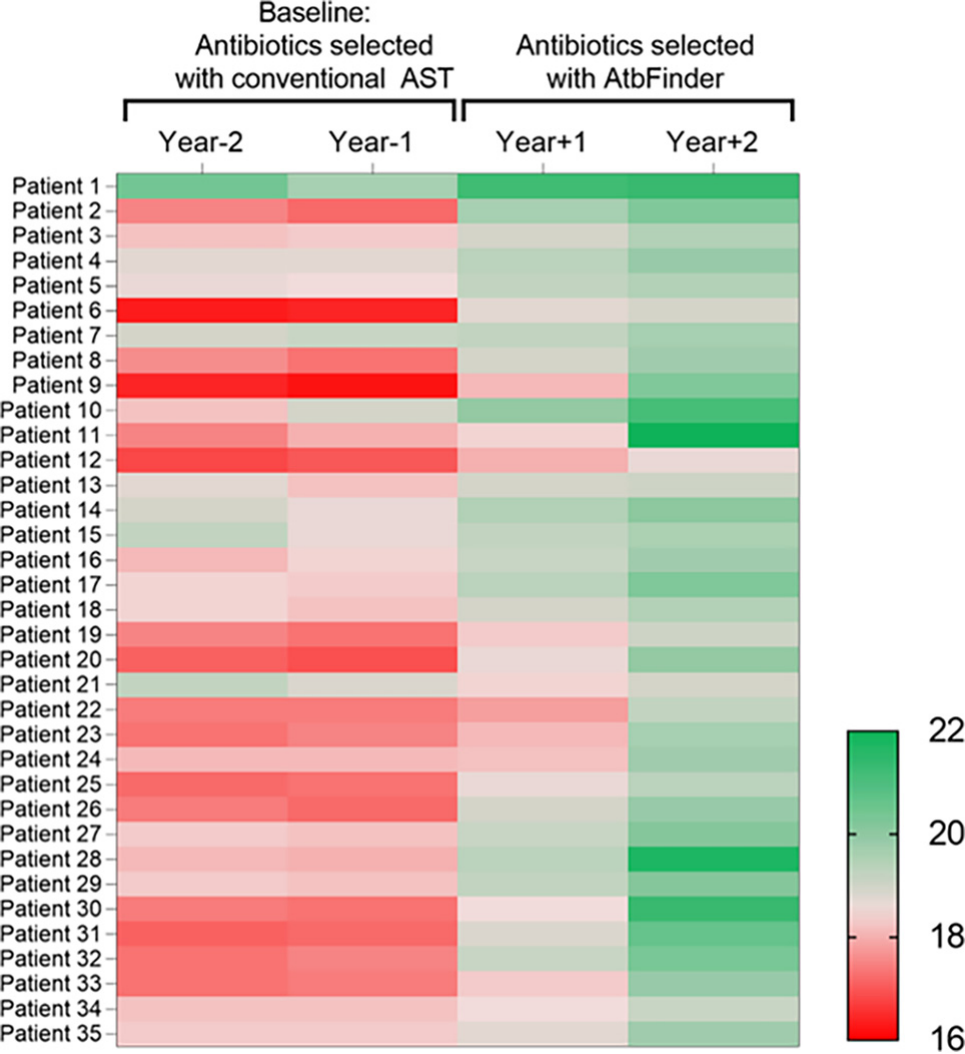
Heat map of BMI at baseline and after use of antibiotics selected with AtbFinder. The color intensity in each panel shows the percentage in a sample, referring to color key at the bottom. The BMI is denoted by using a gradient from red (underweight) to green (normal weight).

Changes in BMI after the use of antibiotics selected with AtbFinder were compared with years −2 and −1, which were taken as the baseline period. At baseline, the mean BMI was 17.9 (range, 16.2 to 20.4), which is categorized as “underweight.” At year −1, 27 patients had a BMI of <18.5, of which 11 had a BMI of <17.5; thus, 16 were classified as very underweight (Fig. 6).

By year +1, the use of antibiotics selected with AtbFinder contributed to a mean weight gain of 0.98 BMI unit (*P* < 0.001) and 1.99 BMI unit (*P* < 0.001) by year +2 compared to year −1 (table S5). Notably, after the first year of antibiotic use selected with AtbFinder, 18 of the 27 (66.7%) who were underweight at baseline were of normal weight. By the second year of a new antibiotic regimen, all (100%) patients had a normal weight ranging from 18.6 to 22.0%.

### Evaluation of AtbFinder use for the selection of antibiotics within 4, 8, and 24 h.

Finally, we evaluated the performance of AtbFinder by comparing the time of the appearance of visible bacterial growth at 4 h, 8 h, and 24 h after plating of biological samples on antibiotic-free TGV agar and comparing these results with those grown on AtbFinder with the wells filled with LB or Columbia agar. Since no patients with B. cepacia infection were enrolled in the prospective study, due to the high medical importance of this pathogen, we additionally included bacteria of the BCC from five biological samples with previously confirmed BCC and compared the time of the appearance of visible bacterial growth on AtbFinder filled with antibiotic-free TGV agar and Burkholderia cepacia-selective agar.

**FIG 7:**
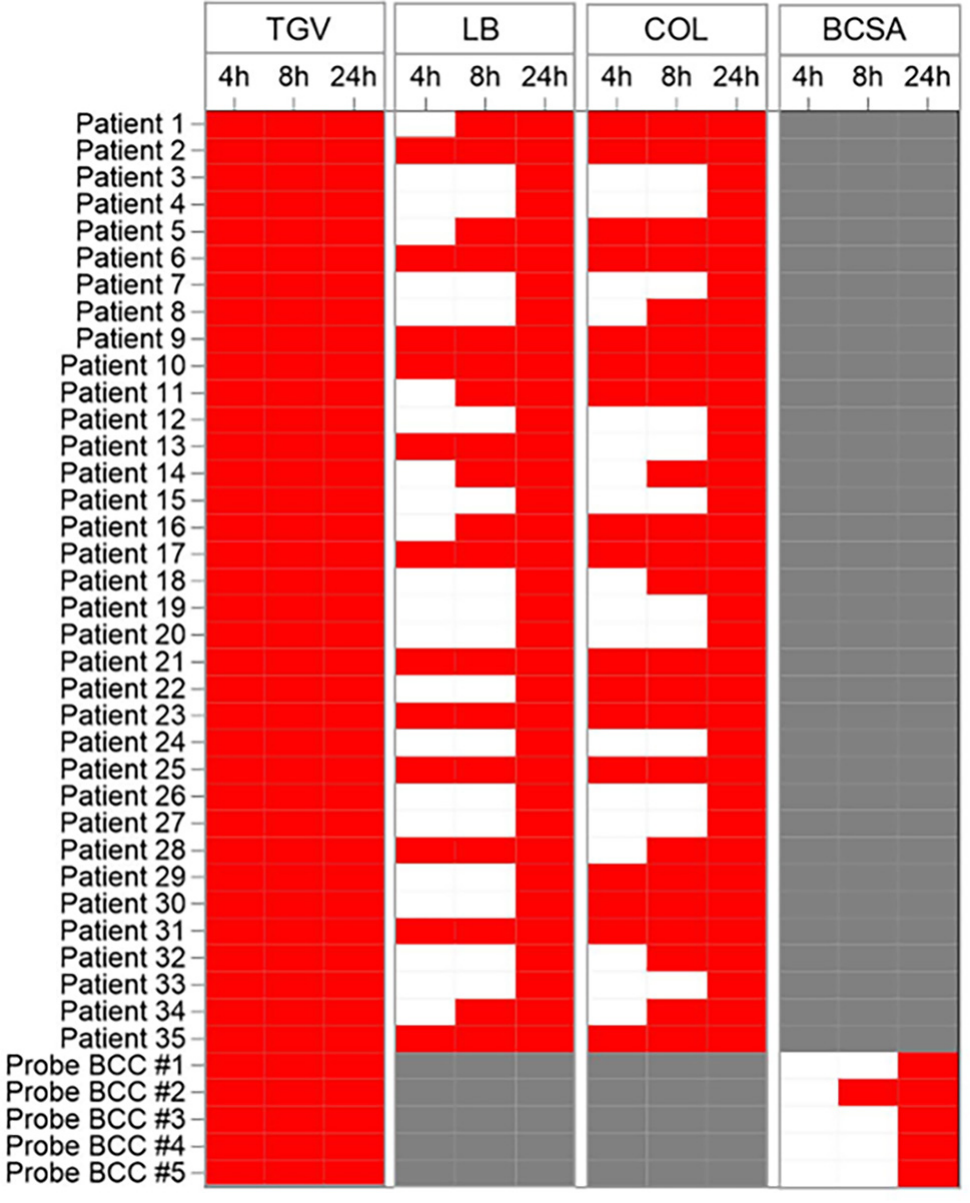
Comparative analysis of bacterial growth rate in different media after direct plating of biological specimen. Growth rate is represented by a heat map, with each cell indicating the presence of microbial growth at a certain time period on different media. TGV, TGV agar; LB, LB agar; COL, Columbia agar; BCSA, Burkholderia cepacia-selective agar. Red color represents the presence of visible microbial growth, whereas white color indicates no growth for a particular time period. Gray color is used for the probes not tested on a certain media.

We found that visible growth on TGV agar was detected in 35 out of 35 (100%) samples already after 4 h (Fig. 7). Since BCC tends to grow slowly and is easily obscured by overgrowth of other microorganisms present in biological samples, we performed initial cultivation on Burkholderia cepacia-selective agar with the follow-up subculturing on TGV agar to and confirmed the growth of BCC in 5 out of 5 (100%) samples within 4 h.

Within the studied time periods, visible growth on other agar media was detected in fewer biological samples. Growth on LB and Columbia agar for 4 h resulted in visible growth of only 12/35 (34.3%) and 17/35 (48.6%), respectively. An additional cultivation time of up to 8 h led to the appearance of visible microbial growth on 18/35 (51.4%) and 23/35 (65.7%) samples on LB and Columbia agar, respectively. Burkholderia cepacia-selective agar, which was used only for the growth of five samples with confirmed BCC, enabled the growth of zero biological samples at 4 h (0%) and 1/5 (20%) at 8 h. By 24 h of growth, visible bacterial growth was observed for all probes on any medium. The data indicated that TGV agar enabled faster visible growth of the biological samples, including isolated B. cepacia, compared to other media.

## DISCUSSION

Our results represent the first study to evaluate the clinical performance of AtbFinder system based on a novel approach by selecting antibiotics that account for the polymicrobial nature of infections. The approach utilized in AtbFinder, which tests the effect of antibiotics against the entire population of microorganisms, is strikingly different from broadly used conventional susceptibility testing wherein the efficacy of antibiotics is evaluated solely against the lead pathogen. We have demonstrated that the antibiotics selected using AtbFinder among persons with CF were more effective in terms of clinical and microbiological outcomes than the antibiotics selected for the same study participants using routine ASTs. Antibiotics selected using AtbFinder enabled a dramatic reduction in the use of systemic antibiotics.

Our findings are in agreement with the latest publications suggesting that new principles of antibiotic selection are required to facilitate optimal patient treatment (2, 3). This is believed to occur primarily due to the methodology of conventional AST as antibiotic susceptibility of the lead pathogen in isolated culture is different from its susceptibility at the site of infection (14, 25).

AtbFinder utilizes a novel TGV nutrient medium, which enables the cultivation of a broader diversity of bacteria from the biological sample. Although no *in vitro* test can perfectly replicate what happens at the infection site, the formation of mixed microbial population on TGV agar allows bacteria to support each other’s growth and enables closer representation of the growth conditions within the host environment including cell-to-cell interaction via newly discovered DNA- and RNA-based TezR receptors (17, 44–46). Using TGV media, we were able to cultivate P. aeruginosa in 15.2% of patients who were previously believed to be P. aeruginosa free. This discrepancy can be explained by the observations of previous studies that the presence of P. aeruginosa is frequently missed by culture with a standard nutrient medium due to low viable counts in a biological sample and inocula (47). Additionally, AtbFinder cultures specimens in the form of mixed community rather than in isolated cultures.

Another critical difference in the novel approach used by AtbFinder compared with routine AST is that the antimicrobial selection by AtbFinder is not based on MIC evaluation. A significant limitation of antibiotic selection based on MIC is that established MIC-based thresholds of bacterial sensitivity to antibiotics are based on antibiotic concentrations that are attainable in the bloodstream (34, 48). This does not take into consideration the particularities of the pharmacokinetics of antibiotics in different tissues. In contrast, antibiotic selection with AtbFinder is based on the concentrations of antibiotics achieved at the site of infection, thus better reflecting the interaction between bacteria and antibiotics within the host. One of the central findings of the present study is that, once antibiotic regimens were switched to those selected with AtbFinder, P. aeruginosa could be cleared in patients along with an increase in FEV1%. We describe the successful clearance of P. aeruginosa from airway cultures in 81.8% of patients treated with antibiotic regimens selected using AtbFinder. Importantly, clearance was not associated with the escalation of antibiotic therapy. In fact, the number of systemic antibiotic courses decreased from 70 to 45 during the first year of AtbFinder usage in patients in whom these antibiotics led to clearance of P. aeruginosa. Additionally, there was a reduction in the need for combination systemic antibiotic therapy with beta-lactam plus another drug class.

This could potentially be a change in therapy of persons with CF, who are today treated with two antipseudomonal antibiotics to enhance activity, despite the fact that the question of monotherapy versus combination therapy has not been clearly validated (49).

Based on the findings of this study the improved outcomes were not associated with the age of the patients or the duration of P. aeruginosa colonization. Other significant improvements were made, including increased predicted FEV1% and decreased the number of hospitalizations. Importantly, our study revealed a significant improvement in lung function following the implementation of antibiotic regimens selected by AtbFinder regardless of the severity of their lung disease (50). These results are surprising given that halting further FEV1% decline in patients with more advanced lung disease is infrequently seen (51).

Antibiotics selected using AtbFinder decreased the need of hospitalization due to pulmonary exacerbation in all study participants, including those who had previously been admitted a few times in a year. Additionally, inflammatory markers that correlate with lung injury were significantly decreased, indicating a reduction in inflammation in these patients that is consistent with other clinical outcomes. Finally, with the antibiotics selected with AtbFinder, we were able to achieve an improvement in BMI in patients with baseline values that were lower than normal levels.

This study has certain limitations, including the small sample size and the fact that we assessed a single group of patients who served as their own controls based on their history. However, the course and constant progression of CF are well described. Moreover, these patients received therapy within the same hospital settings, meaning that, from a medical-care perspective, nothing except the antibiotic therapy was changed.

An additional limitation of this study is the lack of bronchoalveolar lavage (BAL) fluid analysis, which is considered the gold standard for microbiological analysis in persons with CF. However, due to the relative invasiveness of BAL fluid analysis, some authors point to its disadvantage as it contributes additional heterogeneity to microbiological content of biological specimens (52). Therefore, we do not think that these limitations significantly affected the results of this study that aimed to evaluate the novel approach of AtbFinder. Future studies with a larger number of persons with CF and control groups will be necessary to uncover the full potential of the clinical efficacy of AtbFinder.

Data from this study support the hypothesis that the antibiotics selected based on a novel principle of population response are clinically more effective than conventional routine AST methods. Furthermore, when comparing time to results, the AtbFinder system, which enables direct sampling of biosamples without the need for time-consuming pure bacterial culture isolation, required only 4h to select antibiotics with AtbFinder, while it took 48 h for routine phenotypic AST to do so. The data from this study demonstrate that AtbFinder, with as little as 4-h turnaround time, can improve net health outcomes by the selection of more effective antibiotics compared to conventional therapeutic approaches. Additional clinical uses and value may reside by the use of additional variations of the AtbFinder for selecting antibiotic therapies for other pulmonary as well as nonpulmonary infections when AtbFinder tests antibiotics used to treat these indications at the respective concentrations that could be achieved at the particular site of infection.
